# EARDS: EfficientNet and attention-based residual depth-wise separable convolution for joint OD and OC segmentation

**DOI:** 10.3389/fnins.2023.1139181

**Published:** 2023-03-09

**Authors:** Wei Zhou, Jianhang Ji, Yan Jiang, Jing Wang, Qi Qi, Yugen Yi

**Affiliations:** ^1^College of Computer Science, Shenyang Aerospace University, Shenyang, China; ^2^School of Software, Jiangxi Normal University, Nanchang, China; ^3^Shenyang Aier Excellence Eye Hospital Co., Ltd., Shenyang, China; ^4^Party School of Liaoning Provincial Party Committee, Shenyang, China

**Keywords:** glaucoma, joint optic disc and cup segmentation, EfficientNet, attention, residual depth-wise separable convolution

## Abstract

**Background:**

Glaucoma is the leading cause of irreversible vision loss. Accurate Optic Disc (OD) and Optic Cup (OC) segmentation is beneficial for glaucoma diagnosis. In recent years, deep learning has achieved remarkable performance in OD and OC segmentation. However, OC segmentation is more challenging than OD segmentation due to its large shape variability and cryptic boundaries that leads to performance degradation when applying the deep learning models to segment OC. Moreover, the OD and OC are segmented independently, or pre-requirement is necessary to extract the OD centered region with pre-processing procedures.

**Methods:**

In this paper, we suggest a one-stage network named EfficientNet and Attention-based Residual Depth-wise Separable Convolution (EARDS) for joint OD and OC segmentation. In EARDS, EfficientNet-b0 is regarded as an encoder to capture more effective boundary representations. To suppress irrelevant regions and highlight features of fine OD and OC regions, Attention Gate (AG) is incorporated into the skip connection. Also, Residual Depth-wise Separable Convolution (RDSC) block is developed to improve the segmentation performance and computational efficiency. Further, a novel decoder network is proposed by combining AG, RDSC block and Batch Normalization (BN) layer, which is utilized to eliminate the vanishing gradient problem and accelerate the convergence speed. Finally, the focal loss and dice loss as a weighted combination is designed to guide the network for accurate OD and OC segmentation.

**Results and discussion:**

Extensive experimental results on the Drishti-GS and REFUGE datasets indicate that the proposed EARDS outperforms the state-of-the-art approaches. The code is available at https://github.com/M4cheal/EARDS.

## 1. Introduction

Glaucoma is an eye disease that becomes the first leading cause of irreversible vision loss in the world (Weinreb et al., 2014; Mary et al., 2016). It is estimated that 111.8 million people will suffer from glaucoma by the year 2040 (Tham et al., 2014). Since the visual field loss is not evident (Giangiacomo and Coleman, 2009) at an early stage of glaucoma, the damage to visual function is progressive and irreversible when patients are diagnosed with glaucoma. Hence, early-stage glaucoma screening is critical.

**FIGURE 1:**
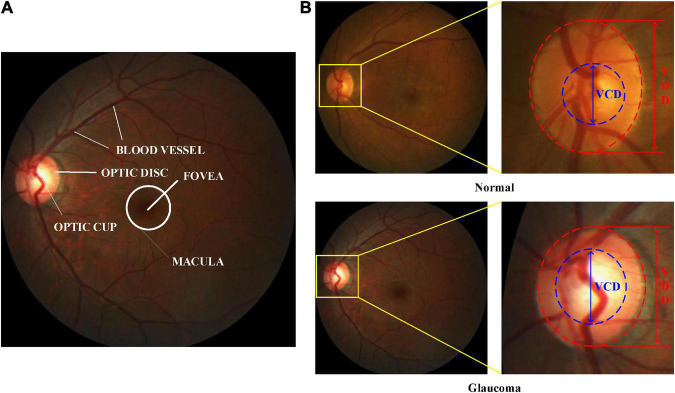
Color retinal fundus images. **(A)** Main structures in a color retinal fundus image. **(B)** VCD and VDD in the normal and glaucomatous color retinal fundus images.

At present, retinal color fundus image plays the most widely used imaging technique at early-stage glaucoma screening, due to cost-effective. In a color retinal fundus image, it has various retinal structures, e.g., Optic Disc (OD), Optic Cup (OC), blood vessels, macula, and fovea, as depicted in Figure 1A. Figure 1B illustrates the vertical OC to OD ratio denoted as CDR, which is well accepted and the prime attribute in glaucoma screening (Fernandez-Granero et al., 2017). CDR can be calculated by the ratio of the Vertical Cup Diameter (VCD) to the Vertical Disc Diameter (VDD). If the CDR value is greater than 0.5, then it reports as glaucoma (Soorya et al., 2019). Since the calculation of CDR depends on precise segmentation of OD and OC, manually segmenting these regions always suffers from the following challenges, e.g., lacking qualified ophthalmologists, inter-individual variability of reading and times-consuming (Pachade et al., 2021). Hence, automatic OD and OC segmentation is more suitable for extracting the useful features for glaucoma screening.

Recently, a series of automatic OD and OC segmentation approaches have been developed based on the color retinal fundus images for glaucoma diagnosis, which can be classified into heuristic-based approaches and deep learning-based approaches (Li et al., 2019). For the heuristic-based approaches, they conduct OD and OC segmentation through the handcrafted features, such as color, gradient, and texture features. However, these features belong to artificial feature engineering, which are easily affected by the fundus structures. Hence, their representation capabilities and stability will influence the segmentation performance. Recently, deep learning-based approaches have become the mainstream for research in ophthalmology. Various deep learning-based segmentation approaches have been put forward (Sevastopolsky, 2017; Gu et al., 2019; Sevastopolsky et al., 2019) for accurate segmentation of OD and OC. However, they still face several challenging issues as below: (1) The OD and OC are segmented independently, or pre-requirement is necessary to extract the OD centered region with pre-processing procedures. Hence, they can not only enhance the computational complexity, but also reduce the accuracy of segmentation due to the separate operations. (2) The high redundancy features always contain in the current segmentation models, which may weaken the reliability and accuracy of segmentation. (3) The issue of vanishing gradient will occur as the network depth increases, leading to overfitting. (4) The extreme OD and OC class imbalance issue encountered in the color fundus images especially for healthy eyes will result in large segmentation errors.

To overcome these limitations, this paper designs an end-to-end joint OD and OC segmentation network named EfficientNet and Attention-based Residual Depth-wise Separable Convolution (EARDS). The main contributions of this paper can be summarized as:

(1) The proposed EARDS is a one-stage approach for joint OD and OC segmentation.
(2) RDSC block is proposed to improve the segmentation performance and computational efficiency. A novel decoder is designed by using RDSC, AG, and BN that leads to promote faster convergence, eliminate vanishing gradient problem, and improve segmentation accuracy.
(3) Our approach achieves better performance, compared with the state-of-the-art approaches on two publicly available datasets.

The rest of this paper is organized as follows. Section 2 gives a brief description of the related works. Section 3 presents the proposed approach in detail. Analysis of experimental results will be introduced in Section 4. Section 5 concludes the paper.

## 2. Related works

Recently, most automatic OD and OC segmentation approaches have been put forward. According to different feature engineering techniques, previous studies can be divided into traditional machine learning approaches based on handcraft features and deep learning-based approaches.

### 2.1. Traditional machine learning approaches

In the early stage, traditional machine learning approaches mainly rely on the handcrafted features for OD and OC segmentation, which are divided into three categories (Morales et al., 2013): appearance-based approaches, model-based approaches, and pixel-based classification approaches. For appearance-based approaches, they always detect the OD and OC through the physiological structure in the retinal fundus images, e.g., OD is the brightest circular (Lalonde et al., 2001; Zhu and Rangayyan, 2008) or elliptical object (Cheng et al., 2011). These approaches are further divided into template-based techniques (Aquino et al., 2010), deformable models-based techniques (Xu et al., 2007), morphology-based techniques (Welfer et al., 2010) and principal component analysis (Dai et al., 2017). However, the main limitation in these approaches is that they can hardly represent the OD regions with irregular shape and contours due to the images with more visible pathologies or lower quality. For model-based approaches, they always utilize the position prior knowledge of OD, OC, and blood vessels for OD and OC segmentation. For instance, OD is the convergence region of the major blood vessels (Mahfouz and Fahmy, 2010) and vessel bends can be regarded as the center of OC (Wong et al., 2008). According to these prior knowledges, reference (Hoover and Goldbaum, 2003) first detected the blood vessels and the OD and OC regions can be segmented based on the acquired vessels. Nevertheless, when the image quality is poor or the blood vessels are detected badly, they can hardly work well for OD and OC segmentation. Pixel-based classification approaches regard the OD and OC segmentation as a supervised pixel classification problem. Cheng et al. (2013) designed a superpixel classification approach for OD segmentation. First, the authors aggregated the pixels from the retinal fundus images into superpixels and then divided each superpixel into the OD regions or non-OD regions. In summary, there are two main limitations in the above-discussed segmentation approaches (Li et al., 2020). On one hand, they depend heavily on handcrafted features and lack generalization. On the other hand, they segment the OD and OC in two separate steps and the mutual relation between them is ignored.

### 2.2. Deep learning approaches

Convolutional Neural Networks (CNNs) can automatically extract the complex features from the input images, which have achieved huge achievements in medical image processing especially for segmentation area (Çiçek et al., 2016). Therefore, a series of CNN variants have attempted to perform OD and OC segmentation, which have achieved excellent performance (Maninis et al., 2016; Sevastopolsky, 2017; Zilly et al., 2017; Al-Bander et al., 2018; Fu et al., 2018; Kim et al., 2019; Shah et al., 2019; Yin et al., 2019; Yu et al., 2019; Pachade et al., 2021). For example, Maninis et al. (2016) suggested a DRIU network based on the VGG-16 for segmenting retinal vessels and OD at the same time. However, the more challenging OC boundary cannot be extracted. Inspired by Region Proposal Network (RPN), Yin et al. (2019) proposed a PM-net for OD and OC segmentation where a pyramidal RoIAlign module is designed to capture multi-scale features. In 2015, a series of Fully Convolutional Network (FCN) (Long et al., 2015) based models have been proposed, in which the FCN-based UNet (Ronneberger et al., 2015) is the most advanced and widely utilized model for medical image segmentation. Motivated by the success of UNet, most UNet variants have been presented to segment the OD and OC. For example, Sevastopolsky (2017) suggested a modified UNet for automatic OD and OC segmentation. Unlike the original UNet, the authors adopt fewer filters and a modified loss function, which has the merits of fast processing speed and few parameters. In Zilly et al. (2017), the authors incorporated an entropy-based sampling technique into CNN framework for OD and OC segmentation which has achieved competitive results. First, an entropy sampling technique is employed to extract informative points. Then, the segmentation results can be acquired by graph cut algorithm. However, these approaches segment OD and OC in a sequential way, thus their effectiveness is limited. To address this issue, a series of two-stage joint OD and OC segmentation approaches have been proposed, in which the first stage is to locate the Optic Nerve Head (ONH) area, and the second stage is to segment OD and OC within the extracted ONH area. For example, Al-Bander et al. (2018) designed a U-shape network structure by combining DenseNet with UNet for OD and OC segmentation simultaneously. Fu et al. (2018) proposed a M-net based on UNet, which consists of multiple inputs and multiple outputs for joint OD and OC segmentation. Similarly, Yu et al. (2019) proposed an improved UNet approach by making full use of the advantage of the pre-trained ResNet and UNet to speed up the model and avoiding overfitting. Kim et al. (2019) detected the Region of Interest (ROI) area around OD, followed by the OD and OC segmentation. In their model, FCN with UNet framework is employed to segment the OD and OC from the ROI. Moreover, Shah et al. (2019) proposed a weak ROI model-based segmentation (WRoIM) approach. In WRoIM, it firstly acquires the coarse OD segmentation regions through a small UNet structure and then inputs the coarse segmentation results into another UNet to obtain accurate fine segmentation. Recently, Pachade et al. (2021) introduced the adversarial learning into the OD and OC segmentation tasks, which acquires a remarkable performance. The studies undertaken above, either segment the OD and OC separately (Maninis et al., 2016; Sevastopolsky, 2017; Zilly et al., 2017; Yin et al., 2019) or require extracting the OD centered region with pre-processing procedures (Al-Bander et al., 2018; Fu et al., 2018; Kim et al., 2019; Yu et al., 2019; Pachade et al., 2021). Therefore, their performance and computational cost will be significantly affected.

## 3. The proposed approach

The proposed EARDS a fully automatic end-to-end network for joint OD and OC segmentation. Next, more detailed descriptions of EARDS will be provided.

### 3.1. Network architecture

**FIGURE 2:**
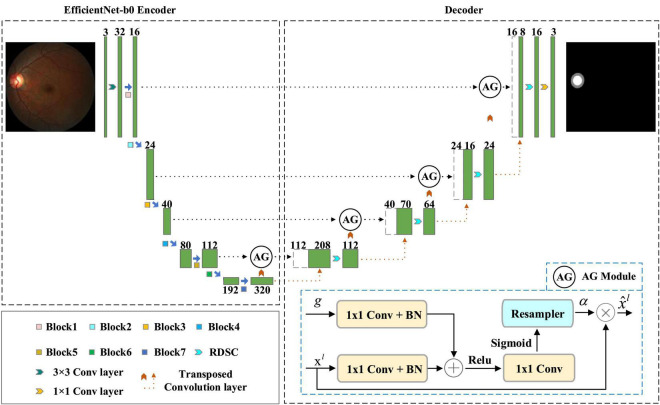
An overview of the proposed EARDS.

The overview of our EARDS is depicted in Figure 2, composed of an encoder-decoder structure. The encoder is a EfficientNet-b0, which is used to extract features from the input fundus images and then convert the features to high-level visual representations. The decoder is a novel network which contains Attention Gate (AG), Residual Depth-wise Separable Convolution (RDSC) block and Batch Normalization (BN) layer. First, we incorporate AG into the skip connection to eliminate the irrelevant regions and highlight features of fine OD and OC regions. Second, to preserve more spatial information from minor details of the OD and OC regions, RDSC block is suggested to replace the traditional convolution operations. The introduction of RDSC is able to achieve the best trade-off between performance and computational efficiency. In addition, BN layer can further eliminate the vanishing gradient problem to accelerate the convergence speed. The final outputs of the decoder network are the segmented OD and OC results. More detailed descriptions are given as below.

#### 3.1.1. EfficientNet

Convolution Neural Networks (CNNs) have been utilized for extracting the key features from the image. Development of a CNNs is done at a fixed resource budget. If there is increase in resources then scaling is done to improve accuracy. A series of ways for scaling CNNs, which can be divided dimension-depth based or width based or image resolution based. Among them, dimension-depth based is widely used. However, due to the tedious manual tuning scaling, it always gives sub-optimal performance. Recently, Tan and Le (2019) research the relationship between width and depth of CNN models and put forward efficient CNN models with less parameters, achieving excellent classification performance. In their study, a baseline model called EfficientNet-b0 is developed, which is scaled up to acquire a family of EfficientNets from B1 to B7. These models have achieved Top-1 accuracy in the ImageNet dataset (Krizhevsky et al., 2017).

**FIGURE 3:**
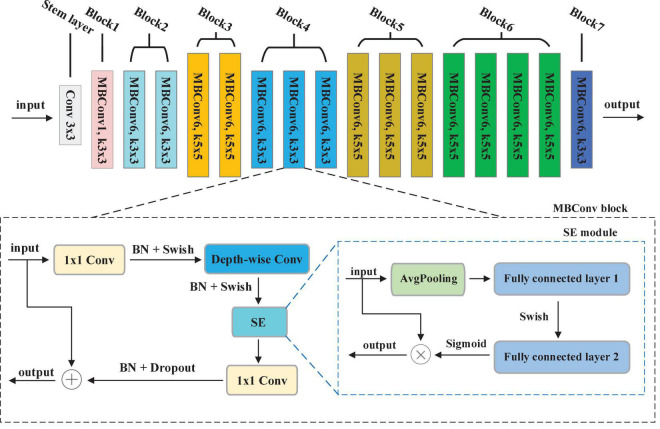
The structure of EfficientNet-b0.

In EfficientNet models, Mobile inverted Bottleneck Convolution (MBConv) is the main building block proposed by Sandler et al. (2018), as depicted in Figure 3. It consists of 1 × 1 convolution (1 × 1 Conv), Depth-wise convolution (Depth-wise Conv) and Squeeze-and-Excitation (SE) module. First, the output of the previous layer is sent to MBConv block and then the number of channels is expended by 1 × 1 Conv. Second, a 3 × 3 Depth-wise Conv is utilized to reduce the number of parameters further. Third, channel pruning reduces number of channels by a 1 × 1 Conv layer. At last, the residual connection between the input and output of the projection layer is introduced. Figure 3 shows the SE module, which contains squeeze operation and excitation operation. First, global average pooling (AvgPooling) is used for squeeze operation. After that, excitation operation is performed which contains two fully connected layers, a Swish activation, and a Sigmoid activation function.

To achieve the best segmentation performance with low resource consumption, this paper chooses the EfficientNet-b0 depieced in Figure 3 as our encoder. The overall structure of EfficientNet-b0 contains 7 MBConvX blocks, represented by different colors. For simplify, we employ ksize to represent the size of convolution kernel, i.e., 3 and 5. The symbol X indicates the coefficient of channel number scaling, e.g., MBConv6 denotes MBConv with a scaling factor of 6. According to the reference (He et al., 2016), EfficientNet-b0 has 5.3M parameters, which is 4.9 times smaller and 11 times faster than ResNet-50. To obtain larger inputs and outputs in the encoding phase, this paper modifies the Stem layer by convolution (kernel = 3, stride = 1, padding = 1).

#### 3.1.2. Attention gate (AG)

Early work on OD and OC segmentation from color fundus image employ two-stage (Maninis et al., 2016; Sevastopolsky, 2017; Zilly et al., 2017; Yin et al., 2019), involving ROI extraction and subsequent segmentation. In particular, these approaches first require extracting the OD centered region with pre-processing procedures and then conduct OD and OC segmentation in sequence. In this way, their computational cost will be significantly affected. Moreover, OC segmentation is more challenging than OD segmentation due to its large shape variability and cryptic boundaries. Therefore, false-positive predictions for OC segmentation remains difficult to reduce. Motivated by the successfully applied of AG in deep learning-based computer vision tasks, this paper introduces AG into our model to locate the most significant features and eliminate redundancy, achieving end-to-end segmentation. Attention Gate proposed by Oktay et al. (2018) belongs to attention mechanism, which allows the model to adaptively adjust and automatically learn the highlight salient features from an input image. Figure 2 shows the structure of AG, in which *g* and *x^l^* are the input feature maps sampled from the current layer and the previous layer, respectively. Based on these feature maps, performing a group of operations include 1 × 1 convolution (1 × 1 Conv), BN, and a point-by-point summation operation. After that, the attention coefficient α can be obtained by executing a series of operations (Rectifier Linear Unit (ReLU) activation + 1 × 1 Conv + Sigmoid activation + resampler operation) in turn. Finally, the final output feature map ${\hat{x}}^{l}$ can be acquired by multiplying the attention coefficient α with the input feature map *x^l^*.

Since AG can be linearly transformed without any spatial support and the resolution of the input feature map will be reduced by down-sampling to the gated signal, the parameter and computational resource of the network model are greatly reduced. Motivated by the advantages of AG, this paper introduces the AG into the original skip connection, which has the following two main merits. For one thing, the promising segmentation performance can be obtained while preserving computational efficiency. For another, the network model can automatically learn the ROI (Region of Interest) implicitly from the original image, eliminating irrelevant regions and focusing on interesting area to be segmented.

#### 3.1.3. Residual depth-wise separable convolution (RDSC)

**FIGURE 4:**
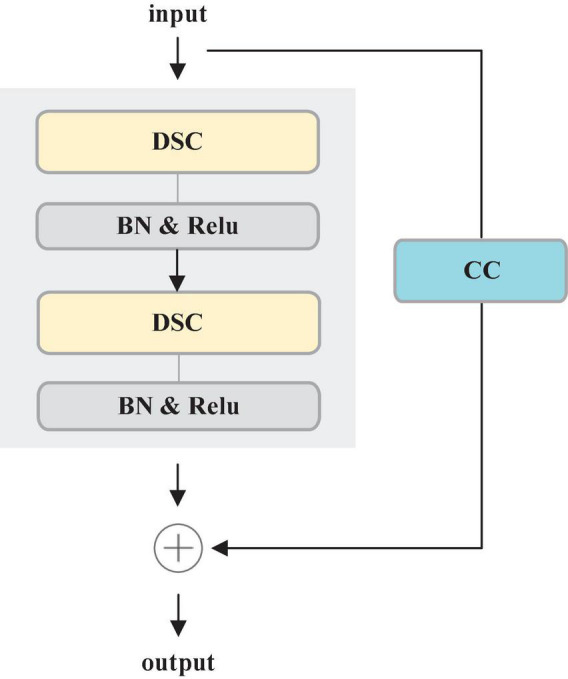
RDSC block.

Most deep learning-based segmentation networks always require large-scaled parameters and high computational cost, leading to hardly deploy the networks to mobile and embedded devices. Moreover, the existing networks tend to overfit the training data. To solve these issues, this paper develops a Residual Depth-wise Separable Convolution (RDSC) block consisting of Depth-wise Separable Convolution (DSC), BN, Rectifier Linear Unit (ReLU) activation and Channel Convolution (CC), as shown in Figure 4.

Each RDSC block contains the following operations: (1) DSC block. (2) BN and ReLU activation are preformed after DSC. (3) CC is to change the number of channels. The main advantages of RDSC are as follows: (1) The network can be deepened and widened without incurring any extra computations. (2) Introducing BN into RDSC speeds up the convergence of the network and effectively avoids the gradient disappearance (He et al., 2016). (3) The nonlinear ReLU activation function increases the nonlinearity of the deep network to learn more complex feature representations.

DSC proposed by Chollet (2017) contains two processes: Depth-wise Convolution and Point-wise Convolution. First, Depth-wise Convolution applies a single filter per input channel. Then, the Point-wise Convolution combines the outputs of Depth-wise Convolution by a 1 × 1 convolution.

Supposing that *F*(*D*_*F*_ × *D*_*F*_ × *M*) and *G*(*D*_*F*_ × *D*_*F*_ × *N*) are the feature maps, where *D*_*F*_ × *D*_*F*_ denotes the spatial width and height. *M* and *N* are the number of input and output channels, respectively. In the standard convolution, the feature map is parameterized as *D*_*K*_ × *D*_*K*_ × *M* × *N* by the convolution with the kernel size (*K*) where *D*_*K*_ is the spatial dimension of the kernel. If stride and padding of convolution are set as 1, the number of parameters of standard convolution is:


(1) $$D_{K}\times D_{K}\times M\times N\times D_{F}\times D_{F}$$

The number of parameters of Depth-wise Convolution is:


(2) $$D_{K}\times D_{K}\times M\times D_{F}\times D_{F}$$

Combining 1 × 1 (point-wise) convolution and Depth-wise Convolution together forms the DSC, and the total number of parameters is:


(3) $$D_{K}\times D_{K}\times M\times D_{F}\times D_{F}+M\times N\times D_{F}\times D_{F}$$

Seen from the above comparisons, the number of parameters of DSC is greatly lower than the standard convolution. When the standard convolution is employed to the proposed model, the parameter size is 17.14 M and the Floating-Point Operations (FLOPs) is 8.51G. Instead, when the RDSC is introduced, it has 15.44 M and 4.21G in parameter size and FLOPs. From these results, we can observe that RDSC can reduce the parameter size and FLOPS by 1.7M and by 4.30G, respectively. Therefore, this paper replaces the standard convolutional blocks with RDSC blocks in the decoding stage.

### 3.2. Loss function

The OD and OC segmentation can be regarded as a multi-class segmentation problem. However, there are two major limitations in the current segmentation approaches. For one thing, the overlapped OD and OC makes the segmentation task more challenging. For another, since the OD and OC regions are much smaller than the background region in the fundus images, the class imbalance issue will influence the model training. Recently, the researchers have proposed dice loss (Milletari et al., 2016) and focal loss (Lin et al., 2017) for the optimization of the parameters, achieving superior performance. Among them, dice loss derived from the dice coefficient reflects the similarity of two contour regions and focal loss is to deal with the class imbalance issue.

Inspired by the advantages of focal loss and dice loss, this paper presents a novel fusion loss function by combining the weighted focal loss and dice loss for joint OD and OC segmentation. The proposed fusion loss function is given as follows:


(4) $$L_{s\,e\,g}\,\left(m,p\right)=L_{D\,L}\,\left(m,p\right)+\lambda \,L_{F\,L}\,\left(m,p\right)$$

where


(5) $$L_{D\,L}\,\left(m,p\right)=1-\sum_{k=1}^{K}\frac{2\,m_{k}\,p_{k}}{{\left(m_{k}\right)}^{2}+{\left(p_{k}\right)}^{2}}$$

$$L_{F\,L}\left(m,p\right)=\sum_{k=1}^{K}[-m_{k}\alpha_{k}{\left(1-p_{k}\right)}^{\gamma }\log p_{k}$$

(6) $$-\left(1-m_{k}\right)\left(1-\alpha_{k}\right){\left(p_{k}\right)}^{\gamma }\log\left(1-p_{k}\right)]$$

where *L*_*DL*_ and *L*_*FL*_ are dice loss and focal loss, respectively. λ is a regularization parameter to balance the weight of *L*_*DL*_ and *L*_*FL*_. *m* ∈ {0,1} is a binary ground truth label, and *p* ∈ [0,1] is the predicted probability value. *K* represents the number of categories, and the proposed weighting factor of the *k*th category is denoted as α_*k*_.

## 4. Experiments and results

### 4.1. Datasets

Extensive experiments are performed on two publicly available datasets, i.e., Drishti-GS (Sivaswamy et al., 2014) and REFUGE (Orlando et al., 2020).

Drishti-GS dataset (Sivaswamy et al., 2014) contains 101 annotated color fundus images, of which 70 and 31 correspond to glaucomatous and normal eyes, respectively. The given split of the dataset contains 50 training images and 51 testing images.

REFUGE dataset (Orlando et al., 2020) consists of 1200 annotated color fundus images, which are equally divided into three subsets of 400 images each to form training, validation, and testing. In the training set, there are 40 glaucomatous images and 360 normal images. In this paper, we adopt the training set to verify the effectiveness of the proposed approach. First, we randomly select 10 glaucomatous images and 30 normal images from the training set forming the testing set. Then, the rest images are regarded as the training and validation sets. We repeat the sample selection process five times, and the averaged result is utilized for performance comparison.

### 4.2. Implementation details

Our approach is implemented based on the PyTorch platform. We carry out the experiments on Windows 10 system with NVIDIA TITAN Xp graphics card with 12 GB of RAM and a single CPU Intel(R) Xeon(R) CPU E5-2620 v4. The network is trained for 30 epochs with a batch size of 2. Root Mean Square Propagation (RMSProp) optimizer is employed with the initial learning rate of 1e-04. The learning rate is automatically decayed by the validation set score, and the loss is automatically adjusted. The values of parameter α in focal loss are set as 0.75, 0.75 and 0.25 for OD, OC, and background, respectively. Meanwhile, the value of tunable parameter γ is set to 2.

To avoid overfitting, this paper performs data augmentation based on the original images to generate new training data. For Drishti-GS dataset, we apply a combination of image horizontal flip, vertical flip, and translation techniques to generate a total of 2,800 images. Similarly, we employ the same data augmentation techniques for REFUGE dataset to generate a total of 2,880 images. All of the images in both datasets are resized to 512 × 512 pixels.

### 4.3. Evaluation metrics

Four widely used performance metrics are adopted to evaluate the effectiveness of the proposed approach, e.g., Dice Coefficients (DC), Jaccard (JAC), CDR Error (CE), and Balance Accuracy (BA).


(7) $$D\,C=\frac{2\times T\,P}{2\times T\,P+F\,P+F\,N}$$

(8) $$J\,A\,C=\frac{T\,P}{T\,P+F\,P+F\,N}$$

(9) $$C\,E=\frac{1}{N}\,\sum_{n=1}^{N}\left|C\,D\,R_{p}^{n}-C\,D\,R_{m}^{n}\right|$$

with


(10) $$C\,D\,R=\frac{V\,D_{c\,u\,p}}{V\,D_{d\,i\,s\,c}}$$

(11) $$B\,A=\frac{1}{2}\,\left(S\,e+S\,p\right)$$

With


(12) $$S\,e=\frac{T\,P}{T\,P+F\,N},S\,p=\frac{T\,N}{T\,N+F\,P}$$

where *TN*, *FN*, *TP*, and *FP* denote the number of True Negatives, False Negatives, True Positives, and False Positives, respectively. $C\,D\,R_{p}^{n}$ is the predicted CDR value of *n*-th image calculated by the segmented result and $C\,D\,R_{m}^{n}$ is the corresponding ground truth CDR from trained clinician. *N* represents the total number of samples in the testing set. Lower the absolute CDR Error value (CE) better is the predicted result. *VD*_*cup*_ and *VD*_*disc*_ are the vertical diameters of OC and OD respectively. *Se* and *Sp* represent sensitivity and specificity.

### 4.4. Experimental results

Extensive experiments are performed to verify the effectiveness of our approach on the Drishti-GS and REFUGE datasets and the acquired experimental results are as below. On the Drishti-GS dataset, our approach achieves the scores of 0.9741, 0.9497, and 0.9745 in terms of DC, JAC, and BA for OD segmentation and it obtains 0.9157, 0.8493, and 0.9205 for OC segmentation, respectively. On the REFUGE dataset, it acquires the scores of 0.9549, 0.9147, and 0.9559 in terms of DC_*OD*_, JAC_*OD*_, and BA_*OD*_. For OC segmentation, the achieved scores are 0.8872, 0.8017, and 0.8957, respectively. To further assist ophthalmologists in diagnosis of glaucoma, the corresponding CDR can be calculated based on the obtained OD and OC segmentation results. We adopt the commonly used CE to evaluate the accuracy of CDR estimation. The results on the Drishti-GS and REFUGE datasets indicate that our approach acquires the scores of 0.0443 and 0.0471 in terms of CE, respectively.

Next, our approach with different loss functions is tested on the REFUGE dataset. In the experiment, cross-entropy loss, dice loss, and focal loss are selected for comparison. Table 1 depicts the segmentation performance with different loss functions.

**TABLE 1:** OD and OC segmentation results by different loss functions on the REFUGE dataset.

Loss function	OD segmentation			OC segmentation			CE
	**DC_OD_**	**JAC_OD_**	**BA_OD_**	**DC_OC_**	**JAC_OC_**	**BA_OC_**	
Cross-entropy	0.9593	0.9225	**0.9600**	0.8815	0.7907	0.8901	0.0518
Dice	0.9557	0.9160	0.9566	0.8911	0.8080	0.9008	0.0542
Focal	0.9528	0.9103	0.9542	0.8702	0.7733	0.8818	0.0580
Cross-entropy+Focal	0.9585	0.9214	0.9592	0.8862	0.8003	0.8952	0.0497
Cross-entropy+Dice	0.9563	0.9172	0.9568	0.8899	0.8053	0.8979	0.0504
**Focal+Dice**	**0.9594**	**0.9226**	**0.9600**	**0.8930**	**0.8100**	**0.9014**	**0.0482**

Bold text indicates the optimal performance.

**FIGURE 5:**
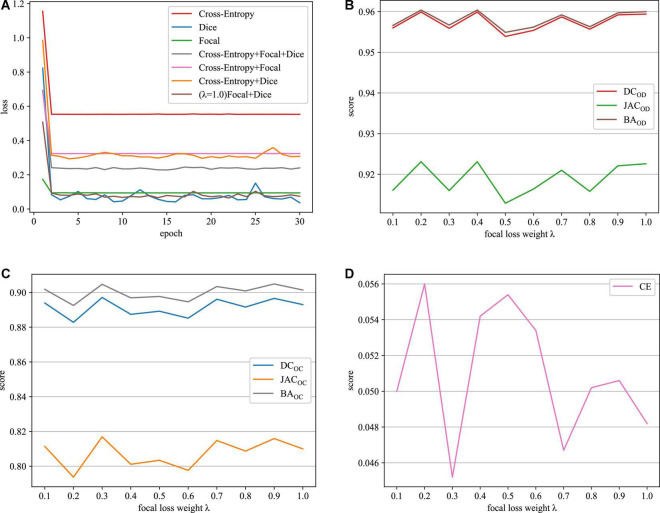
Loss function curves on the REFUGE dataset. **(A)** Training loss curves with different loss functions, **(B)** OD segmentation, **(C)** OC segmentation, and **(D)** CE scores.

As it can be seen in Table 1, when we just employ one kind of loss functions to train model, the cross-entropy loss can always achieve the best performance. Meanwhile, when we combine the cross-entropy loss with dice loss or the focal loss, the segmentation performance will not be further improved. However, appending a focal loss on dice loss constructs the fusion loss for model training, which can achieve the best performance in terms of all the evaluation criteria. Motivated by this, this paper proposes the fusion loss function by incorporating tunable parameters to handle output imbalance. Figure 5A shows loss function curves that are generated from different loss functions. The proposed fusion loss is proved to be more suitable for training network.

The relative weighting λ of the focal loss and dice loss is a major parameter in the proposed fusion loss. In this paper, the role of λ is determined by grid-based searching {0.1, 0.2, 0.3, 0.4, 0.5, 0.6, 0.7, 0.8, 0.9, 1.0}. According to the experiment results depicted in Figures 5B–D, when the value of λ is set to 0.3, our approach can acquire the best results in terms of all the evaluation metrics. Therefore, we recommend λ = 0.3 in the following experiment.

**FIGURE 6:**
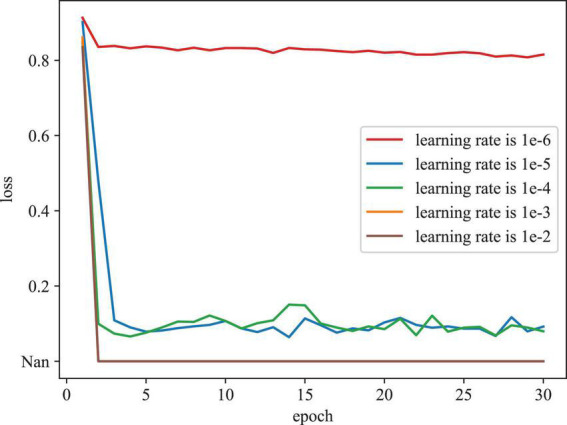
Training loss curves with different initial learning rates on the REFUGE dataset.

To evaluate the effect of various initial learning rates in training model, Figure 6 depicts the training loss curves with different initial learning rates on the REFUGE dataset. As depicted in Figure 6, when the initial learning rate is set as too large (e.g., 1e-2 and 1e-3), the model cannot be trained properly, (denoted as Nan). When the initial learning rate is set as too small (e.g., 1e-06), the model converges slowly and falls into the local optimal point. According to these results, we determine the initial learning rate as 1e-04 in our experiment.

### 4.5. Ablation study

Ablation experiments are conducted on the DRISHTI-GS and REFUGE databases. In our approach, there are three major components including the Efficient-b0, AG module, RDSC. For the sake of description, we utilize the E-UNet, EA-UNet, ERDSC-UNet to represent Efficient-b0 module, Efficient-b0 Attention Gate, and Efficient-b0 RDSC, respectively. The original UNet is regarded as the baseline model and the proposed fusion loss is used to train different components. Meanwhile, the mean DC, JAC, BA and CE are employed to evaluate the segmentation performance. Table 2 summarize the ablation results of OD and OC segmentation on the Drishti-GS and REFUGE datasets, respectively.

**TABLE 2:** OD and OC segmentation results by different models on the Drishti-GS dataset and REFUGE dataset.

Dataset	Model	OD segmentation			OC segmentation			CE
		**DC_OD_**	**JAC_OD_**	**BA_OD_**	**DC_OC_**	**JAC_OC_**	**BA_OC_**	
Drishti-GS	UNet (Baseline)	0.9642	0.9319	0.9654	0.8661	0.7737	0.8845	0.0793
	E-UNet	0.9715	0.9447	0.9719	0.9008	0.8251	0.9083	0.0544
	EA-UNet	0.9572	0.9255	0.9616	0.8888	0.8146	0.9044	0.0513
	ERDSC-UNet	0.9725	0.9467	0.9729	0.9092	0.8395	0.9161	0.0486
	**Our EARDS**	**0.9741**	**0.9497**	**0.9745**	**0.9157**	**0.8493**	**0.9205**	**0.0443**
REFUGE	UNet (Baseline)	0.8849	0.8201	0.9045	0.8258	0.7221	0.8480	0.0821
	E-UNet	0.9521	0.9100	0.9535	0.8838	0.7965	0.8929	0.0503
	EA-UNet	0.9547	0.9141	0.9556	0.8805	0.7908	0.8896	**0.0470**
	ERDSC-UNet	0.9531	0.9118	0.9542	0.8828	0.7942	0.8924	0.0500
	**Our EARDS**	**0.9549**	**0.9147**	**0.9559**	**0.8872**	**0.8017**	**0.8957**	0.0471

Bold text indicates the optimal performance.

**FIGURE 7:**
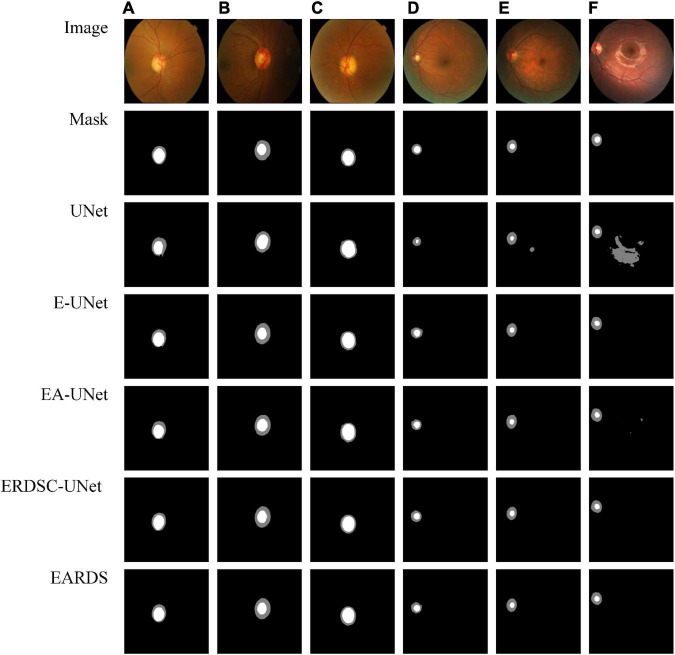
Examples of visual segmentation on the Drishti-GS and REFUGE datasets. **(A–C)** Examples from the Drishti-GS dataset and **(D–F)** examples from the REFUGE dataset.

Seen from Table 2, when the Efficient-b0, AG module, and RDSC block are gradually added into the baseline model, all the evaluation metrics continuedly increase. Hence, the contribution of each improvement in the proposed model is verified and combining these models in a reasonable way can further enhance the segmentation performance. For better visualizing the segmentation results, we select six representative testing images from Drishti-GS and REFUGE datasets, as shown in Figure 7. In Figure 7, the first two rows are original color fundus images and the corresponding ground truth images for OD and OC. The rest 5 rows are the results obtained by different models in ablation study. As observed from Figure 7, each component in our model is effective, and the best segmentation results can be acquired by combining these components together.

**FIGURE 8:**
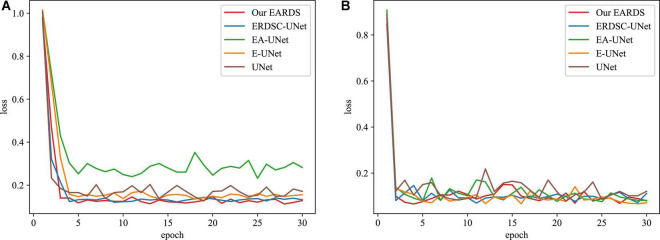
The training loss curves of different models in the different datasets. **(A)** Drishti-GS, **(B)** REFUGE.

Moreover, some main performance evaluation criteria involving the training time, the number of parameters and FLOPS, are also provided in Table 3. As observed from Table 3, the training time will be increased when more components are fused into the baseline model (UNet) and the segmentation performance is enhanced gradually. Apart from the training time, the number of parameters and FLOPS in our model are 1.53M and 3.95G less than EA-UNet, respectively, which can greatly improve the computational cost. All in all, the proposed model best settles the challenging trade-off between segmentation performance and network cost. Figure 8 gives the training loss curves obtained by different models on the Drishti-GS and REFUGE datasets. Seen from these figures, with the increasing number of epochs, the training loss of our model converges with the lowest values, indicating that our model can be successfully trained on the Drishti-GS and REFUGE datasets.

**TABLE 3:** Performance of different ablation models in terms of training time, number of parameters, and FLOPS.

Model	Drishti-GS	REFUGE	Number of parameters	FLOPS
	**Training time**	**Training time**		
UNet	6h 35m 8s	6h 25m 59s	118.40M	218.99G
E-UNet	7h 38m 29s	7h 50m 42s	16.82M	7.71G
EA-UNet	8h 5m 10s	8h 53m 49s	16.98M	8.16G
ERDSC-UNet	8h 14m 40s	8h 49m 5s	15.28M	3.76G
EARDS	9h 26m 40s	9h 26m 23s	15.44M	4.21G

**FIGURE 9:**
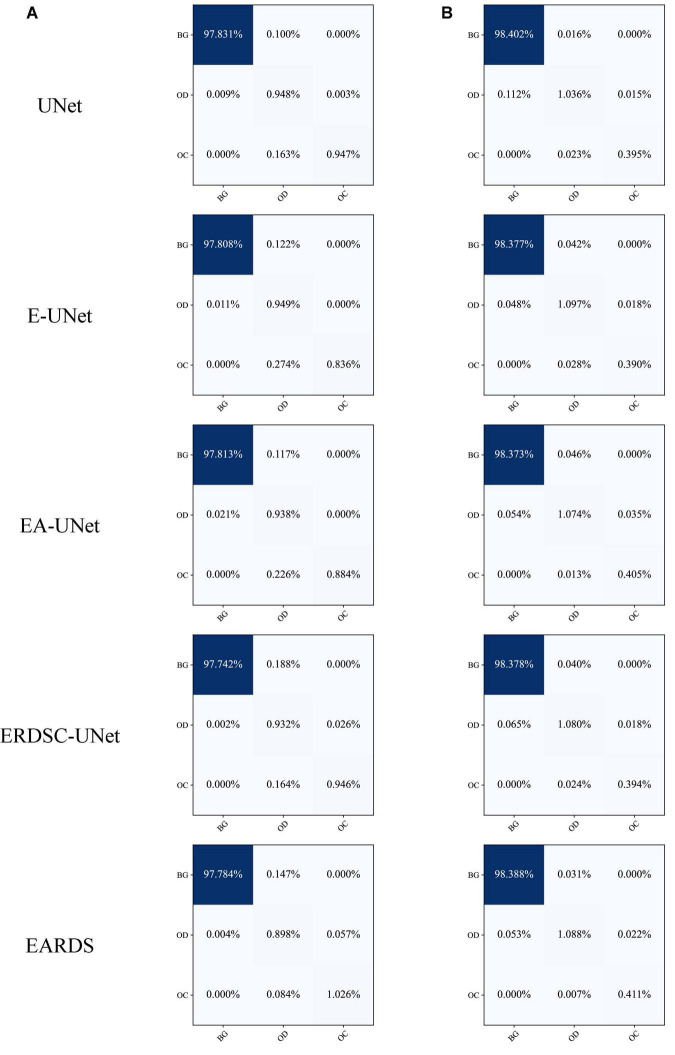
Confusion matrices on different datasets. **(A)** Drishti-GS, **(B)** REFUGE.

Furthermore, the confusion matrices of segmentation results achieved by different models in ablation study are shown in Figure 9. According to the comparison results, we can observe that our model can better distinguish the OD and OC regions from the background, especially for the more challenging OC region. In addition, the number of mis-segmentation pixels in the OD region is lower than that of other models. Hence, the merits of our approach over other models on the segmentation of OD and OC.

**FIGURE 10:**
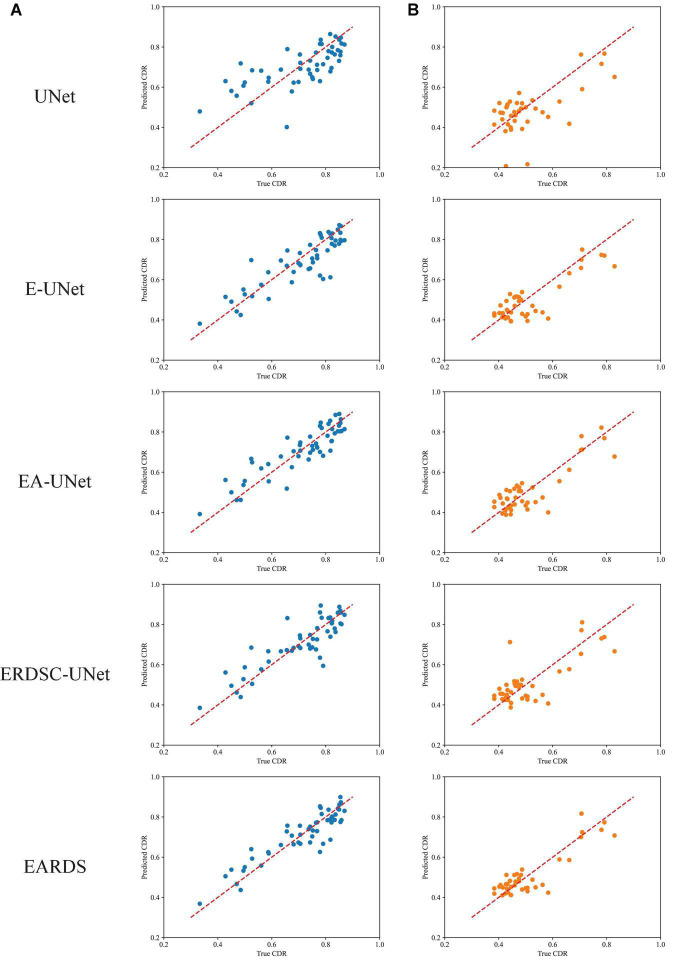
Scatter plots of CDR for different datasets. **(A)** Drishti-GS, **(B)** REFUGE.

Finally, we also calculate the CDR values based on the obtained segmentation results in the ablation study. Figure 10 shows the scatter plots of the corresponding CDR values. As can be observed from Figure 10, the value of CDR calculated by our model is closer to the real CDR. For example, on the Drishti-GS dataset, the scores of UNet, EA-UNet, ERDSC-UNet and our model are respectively 0.0793, 0.0513, 0.0486, and 0.0443 in terms of CE. On the REFUGE dataset, UNet, EA-UNet, ERDSC-UNet and our model achieve 0.0821, 0.0470, 0.0500, 0.0471 in CE, respectively. Compared with these models in the ablation study, our model obtains higher accuracy on CDR calculation.

### 4.6. Glaucoma screening

**FIGURE 11:**
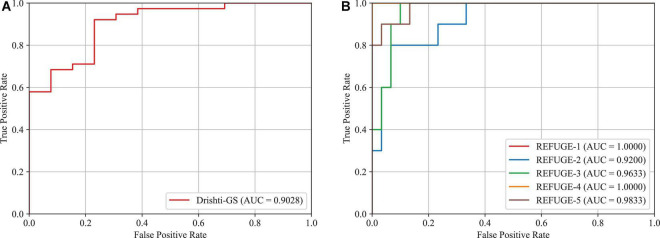
AUC scores and ROC curves for glaucoma screening based on CDR. **(A)** Drishti-GS, **(B)** REFUGE.

In this subsection, we will validate the effectiveness of the proposed approach in glaucoma screening. Since the vertical CDR is an important metric for glaucoma screening, we calculate it via the obtained OD and OC segmentation masks. This paper adopts the Receiver Operating Characteristic (ROC) curve and Area Under the ROC Curve (AUC) as the metrics. The results of glaucoma screening on the Drishti-GS and REFUGE datasets are depicted in Figures 11A, B, respectively. Since the REFUGE dataset is tested on five cross-validation datasets separately, there are five ROC curves as shown in Figure 11B. The averaged AUC score is regarded as the final AUC score. As seen from these figures, the AUC scores obtained by the proposed approach are 0.9028 and 0.9733 on the Drishti-GS and REFUGE datasets, respectively. As seen from these figures, the AUC scores obtained by the proposed approach are 0.9028 and 0.9733 on the Drishti-GS and REFUGE datasets, respectively. According to the reference (Pachade et al., 2021) proposed by Pachade et al., the acquired AUC scores are 0.8968 and 0.9644 on the Drishti-GS and REFUGE datasets, which is lower than our approach. Hence, the proposed approach has a strong potential for glaucoma screening.

### 4.7. Discussion and comparison with the state-of-the-art approaches

In this subsection, we compare the proposed approach with the state-of-the-art approaches, including UNet (Ronneberger et al., 2015), FC-DenseNet (Al-Bander et al., 2018), Yu et al. (2019), WRoIM (Shah et al., 2019), M-Net (Fu et al., 2018), WGAN (Kadambi et al., 2020), pOSAL (Wang et al., 2019), GL-Net (Jiang et al., 2019), CFEA (Liu et al., 2019), Multi-model (Hervella et al., 2020), Two-stage Mask R-CNN (Almubarak et al., 2020), ResFPN-Net (Sun et al., 2021) and M-Ada (Hervella et al., 2022). Table 4 illustrate the OD and OC segmentation results of different approaches on the Drishti-GS and REFUGE datasets, respectively.

**TABLE 4:** OD and OC segmentation results of the state-of-the-art approaches on the Drishti-GS dataset and REFUGE dataset.

Dataset	Methods	OD segmentation		OC segmentation		CE
		**DC**	**JAC**	**DC**	**JAC**	
Drishti-GS	UNet (Ronneberger et al., 2015)	0.9500	–	0.8200	–	–
	FC-DenseNet (Al-Bander et al., 2018)	0.9490	0.9042	0.8282	0.7113	–
	Yu et al. (2019)	0.9738	0.9492	0.8877	0.8042	–
	WRoIM (Shah et al., 2019)	0.9600	–	0.8900	–	–
	M-Net (Fu et al., 2018)	0.9590	–	0.8660	–	–
	WGAN (Kadambi et al., 2020)	0.9540	–	0.8400	–	0.0860
	pOSAL (Wang et al., 2019)	0.9650	–	0.8580	–	0.0820
	GL-Net (Jiang et al., 2019)	0.9710	–	0.9050	–	–
	Multi-model (Hervella et al., 2020)	0.9607	0.9243	0.9029	0.8229	–
	ResFPN-Net (Sun et al., 2021)	**0.9759**	–	0.8961	–	–
	M-Ada (Hervella et al., 2022)	0.9718	–	0.9103	–	**0.0413**
	**Ours**	0.9741	**0.9497**	**0.9157**	**0.8493**	0.0443
REFUGE	M-Net (Fu et al., 2018)	0.9436	–	0.8315	–	–
	pOSAL (Wang et al., 2019)	0.9460	–	0.8750	–	0.0510
	CFEA (Liu et al., 2019)	0.9416	–	0.8627	–	0.0481
	Multi-model (Hervella et al., 2020)	–	**0.9225**	–	0.7902	–
	Two-stage Mask R-CNN (Almubarak et al., 2020)	0.9477	–	0.8546	–	0.0425
	M-Ada (Hervella et al., 2022)	**0.9585**	–	0.8825	–	**0.0373**
	**Ours**	0.9549	0.9147	**0.8872**	**0.8017**	0.0471

‘–’ Means that there is no performance reported and bolded values denote the best performance of models.

Considering that our approach is an improved structure based on UNet, we first compare it with the original UNet on the Drishti-GS and REFUGE datasets. According to Table 4, it is noteworthy that our approach greatly outperforms the original UNet in terms of DC scores. In addition, some UNet based variants, i.e., M-Net (Fu et al., 2018), FC-DenseNet (Al-Bander et al., 2018), Yu et al. (2019), WRoIM (Shah et al., 2019) are used for performance comparison. As can be seen from Table 4, our approach remarkably performs better than the earlier best result by Yu et al. (2019) on OC DC by around 2.8%. Also, we have higher DC scores of 0.0113 and 0.0557 than M-Net (Fu et al., 2018) for OD and OC segmentation on the REFUGE dataset. Since deep learning approaches based on Generative Adversarial Networks (GAN) have also achieved satisfactory OD and OC segmentation results, some state-of-the-art GAN-based approaches such as CFEA (Liu et al., 2019), pOSAL (Wang et al., 2019), WGAN (Kadambi et al., 2020), and GL-Net (Jiang et al., 2019) are employed to compare. As observed from Table 4, our approach achieves the best performance in terms of all the evaluation metrics on the two datasets. Finally, the proposed approach is compared with the latest deep learning approaches, i.e., Multi-model (Hervella et al., 2020), Two-stage Mask R-CNN (Almubarak et al., 2020), ResFPN-Net (Sun et al., 2021) and M-Ada (Hervella et al., 2022). According to the results, we can learn that the OD segmentation performance of our approach is slightly lower than ResFPN-Net by 0.0018 (DC) on the Drishti-GS dataset and is inferior to M-Ada by 0.0036 (DC) on the REFUGE dataset. However, the OC segmentation is a more challenging and far more complicated than OD segmentation. Under this circumstance, our approach can achieve the best OC segmentation performance.

Among all the comparison approaches, our approach can greatly improve the accuracy of the more challenging OC segmentation and obtain competitive results on the OD segmentation. The main reasons are as below:

1. Our approach directly outputs the segmentation result based on the original color retinal fundus images. Therefore, it cannot only reduce the complexity, but also take the relationship between OD and OC into consideration, which is helpful for OD and OC segmentation.
2. A novel decoder network using AGs, RDSC block and BN layer is suggested to eliminate the vanishing gradient problem and accelerate the convergence speed.
3. To deal with the class imbalance issue in the color retinal fundus images, this paper designs a novel fusion loss function by weighted fusing focal loss and dice loss to train model, which can effectively improve the segmentation performance.

## 5. Conclusion and future work

This paper proposes an end-to-end joint OD and OC segmentation approach. First, we employ the EfficientNet-b0 as an encoder to increase the output feature map size and the feature representation capability. Then, the AG module is applied into the skip connection to suppress the irrelevant regions and highlight the ROI region for OD and OC segmentation. Next, we design a RDSC block to improve the segmentation performance and computational efficiency. Furthermore, taking AG, RDSC and BN into a united framework, a novel decoder network is presented to eliminate the vanishing gradient problem and speed up the convergence speed. Finally, to solve the class imbalance problem in the OD and OC segmentation tasks, a novel fusion loss is proposed. We conduct the proposed approach on the Drishti-GS and REFUGE datasets, which achieves the state-of-the-art performance. In addition, based on the obtained OD and OC segmentation results, the CDR value can be calculated to assess the risk of glaucoma. The results indicate that the proposed approach has a good potential in glaucoma screening.

Although the proposed approach can achieve encouraging performance on the OD and OC segmentation tasks, a challenging problem in our approach is the domain shift, i.e., unstably diagnosis results will be achieved without re-training. Therefore, the domain adaptation will be incorporated into our model to improve its generalization and stability in the future.

## Data availability statement

The datasets presented in this study can be found in online repositories. The names of the repository/repositories and accession number(s) can be found below: REFUGE: https://refuge.grand-challenge.org and Drishti-GS: http://cvit.iiit.ac.in/projects/mip/drishti-gs/mip-dataset2/Home.php.

## Author contributions

WZ, JJ, and YJ: data curation, funding acquisition, methodology, supervision, writing—original draft, and writing—review and editing. YY, YJ, and QQ: data curation and methodology. JW, JJ, and YY: data curation, formal analysis, supervision, and writing—review and editing. All authors contributed to the article and approved the submitted version.
